# Study of clinical correlation of motion sickness in patients with vestibular migraine

**DOI:** 10.3389/fnins.2022.986860

**Published:** 2022-09-20

**Authors:** Danyang Meng, Xuyou Zhou, Tianye Hu, Jialian Zheng, Tingyu Jin, Han Gao, Jin Hu

**Affiliations:** ^1^Department of Neurology, Affiliated Hospital of Jiaxing University, Jiaxing, China; ^2^Department of Traditional Chinese Medicine and Acupuncture, Affiliated Hospital of Jiaxing University, Jiaxing, China; ^3^Department of Physical Examination Center, Affiliated Hospital of Jiaxing University, Jiaxing, China

**Keywords:** vestibular migraine, motion sickness, vertigo, clinical diagnosis, correlation

## Abstract

**Objective:**

In this study, clinical data from vestibular migraine (VM) patients and healthy control populations were collected to analyze the clinical data of VM patients, especially the history of motion sickness, and to understand their clinical characteristics.

**Methods:**

According to VM diagnostic criteria, 140 patients diagnosed with confirmed VM (cVM) and probable VM (pVM) who attended the outpatient and inpatient ward of Jiaxing First Hospital between August 2017 and June 2021, as well as 287 healthy check-ups in the health management center, were analyzed and compared in terms of age, gender, and previous history of motion sickness.

**Results:**

A comparison of clinical data related to VM patients and the control population showed that there were more women in the VM group (*P* < 0.01) and that patients in the VM group were older (*P* < 0.05) and had a higher prevalence of history of motion sickness history (*P* < 0.01). Analysis after matching gender and age revealed that patients in the cVM group were older than those in the pVM group (*P* < 0.05), but the proportion of motion sickness was lower than in the pVM group (*P* < 0.05). The age of the patients in the cVM group was mainly distributed around 50 years of age, following a normal distribution, whereas the age distribution of the patients in the pVM group did not have a significant trend of age concentration and was distributed at all ages.

**Conclusion:**

The history of motion sickness is significant in patients with VM and may be a potential suggestive factor for the diagnosis of VM.

## Introduction

Vestibular migraine (VM) is the second most common cause of recurrent vertigo or dizziness and is clinically second to benign paroxysmal positional vertigo as a subtype of migraine (Mallampalli et al., 2021; Krishnan and Carey, 2022). The typical vestibular symptoms of VM are spontaneous vertigo, positional vertigo, and head motion intolerance, as well as increased visuomotor sensitivity and spatial disorientation (Zaleski-King and Monfared, 2021; Shen and Qi, 2022).

The Migraine Classification Subcommittee of the International Headache Society (IHS) proposed in 2018 that the diagnosis of VM is based on recurrent vestibular symptoms, a history of migraine, a temporal association between vestibular symptoms and migraine symptoms, and the exclusion of other causes of vestibular symptoms (2018).

The latest proposed diagnostic criteria divide the VM into confirmed VM (cVM) and probable VM (pVM), and the diagnosis is based mainly on the clinical manifestations of the patient, lacking specific examination means and objective indicators. According to this criterion, only about 20% of patients have received a relatively accurate diagnosis (Li et al., 2021; Mallampalli et al., 2021). And the first five episodes of dizziness or vertigo in patients with VM were not diagnosed. Motion sickness is a series of physiological reactions caused by abnormal external exercise stimulation, including nausea, vomiting, cold sweat, dizziness, and other symptoms. In daily life, motion sickness often occurs in the process of taking airplanes, ships, cars, and other vehicles, which affects people’s normal life and work (Koch et al., 2018; Cohen et al., 2019; Keshavarz and Golding, 2022). In this article, we analyze the correlation between VM patients and their history of motion sickness to provide more clinical guidance significance for the early diagnosis of VM.

## Materials and methods

### Ethics statement

The study was approved by the Institutional Review Board of the Affiliated Hospital of Jiaxing University, Jiaxing City. Informed consent was obtained from patients and healthy subjects.

### Study population

One hundred and forty patients with a diagnosis of VM and pVM and 287 people who underwent health checks, who were seen in outpatient clinics and inpatient ward of the Department of Neurology, Affiliated Hospital of Jiaxing University between August 2017 and June 2021, were selected and divided into experimental and control groups. Clinical data such as age, gender, and previous history of motion sickness were collected from both groups and the data were analyzed, summarized and compared.

Inclusion criteria: (1) adults aged 18–80 years; (2) diagnostic criteria for cVM patients and pVM patients met the diagnostic criteria of the International Classification of Headache Disorders, 3rd edition, Olesen et al. (2018) and the BARANY Criteria (Lempert et al., 2012); (3) all patients underwent neurological and neurotological examinations, including an evaluation of nystagmus and assessments of limb ataxia and balance to confirm the absence of a central lesion. Magnetic resonance imaging (MRI) was also conducted for differential diagnosis; and (4) average communication skills for filling out the questionnaire. Exclusion criteria: (1) patients with VM who also had other types of vertigo disorders such as central vertigo, benign paroxysmal positional vertigo, etc.; (2) pregnancy; (3) mental illness; and (4) severe systemic diseases (including hepatic failure, uremia, heart failure, and rheumatic immune diseases).

### Methods

#### The retrospective study designs

The study participants were divided into a VM group and a healthy physical examination group (control group), and the VM group was further divided into two groups: the cVM group and the pVM group. Various clinical data including age, sex, and previous history of motion sickness were collected from all participants. We conducted further screening for the sample by referring to Golding questionnaire (Golding, 2006) to determine the history of motion sickness and the data were presented with or without motion sickness. The calculation of sample size was done using an online sample size calculators.^1^ The sample size used in the present study was appropriate based on the result of sample size calculation (α = 0.05, β = 0.2). This study was approved by the Ethics Committee of the Affiliated Hospital of Jiaxing University.

### Statistical analysis

The measurement data were presented as mean ± standard deviation (*M* ± SD) and the count data was presented as a number. SPSS software (version 25.0) was used for the statistical analysis of the collected data. The count data were compared using the chi-square (χ^2^) test. The measurement data were first tested for normality and data that conformed to a normal distribution were compared using the independent samples *t*-test. *P* < 0.05 was considered statistically significant.

## Results

### Analysis of clinical data from the vestibular migraine and control groups

There were 31 males and 109 females in the VM group and 142 males and 145 females in the control group, respectively. After the Chi-square test, the gender difference between the two groups was statistically significant (χ^2^ = 29.173, *P* < 0.0001, Table 1).

**TABLE 1:** Clinical data of the VM and control groups.

	VM group	Control group	χ^2^/*T*-value	*P*-value
Gender (male/female)	31/109	142/145	29.173	0.0001
Age (years)	46.24 ± 12.88	42.29 ± 14.12	−2.792	0.005
Motion sickness (yes/no)	91/49	66/221	71.408	0.0001

The age of the patients in the VM group was 46.24 ± 12.88 years and the age of the people in the control group was 42.29 ± 14.12 years. Data between the two groups showed a statistical difference in age between the two groups (*t* = −2.792, *P* = 0.005, Table 1).

There were 91 and 66 individuals with a history of motion sickness and 49 and 221 individuals without a history of motion sickness in the VM and control groups, respectively. The results between the two groups were statistically different (χ^2^ = 71.408, *P* < 0.0001, Table 1).

### Analysis of clinical data of the vestibular migraine and control groups after age and gender matching

Since there were statistical differences in age and gender between the VM group and the control group, and the number of people in the control group was much larger than that in the VM group, 84 individuals in the control group were removed to rematch the two groups.

After rematching age and gender, there were 64 men and 142 women in the control group, and the gender difference between the two groups was not statistically significant compared to the VM group (χ^2^ = 3.334, *P* = 0.068, Table 2). The age of the control group after rematching was 42.29 ± 14.12 years, which was not statistically different compared to the control group (*t* = 1.567, *P* = 0.068, Table 2). There were 18 and 188 people with and without a history of motion sickness in the control groups, and it is statistically significant compared to the VM group (χ^2^ = 122.273, *P* < 0.001, Table 2).

**TABLE 2:** Clinical data of the VM and control groups after age and gender matching.

	VM group	Control group	χ^2^/*T*-value	*P*-value
Gender (male/female)	31/109	64/142	3.334	0.068
Age (years)	46.24 ± 12.88	44.48 ± 14.90	1.567	0.068
Dizziness or vertigo (yes/no)	91/49	18/188	122.273	<0.001

### Analysis of clinical data of the confirmed vestibular migraine and probable vestibular migraine groups

**FIGURE 1:**
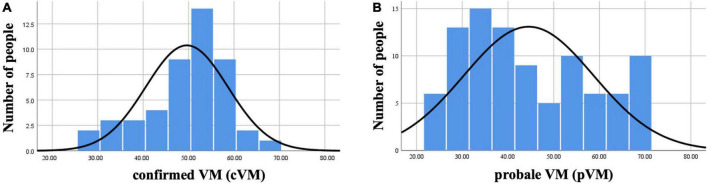
Histogram of the age distribution of VM in the cVM group and the pVM group. **(A)** Histogram of the age distribution of the cVM group. **(B)** Histogram of the age distribution of the pVM group.

After the VM group, the patients were subgrouped into cVM and pVM groups, we performed normality tests for different age groups, and the results showed that the age distribution of the patients in the cVM group was mainly distributed around 50 years of age, which was consistent with a normal distribution, while the age distribution of the patients in the pVM group did not show a significant age concentration trend and was distributed in all age groups (Figure 1).

There were 11 males and 36 females in the cVM group, and 20 males and 73 females in the pVM group, respectively, and the gender difference between the two groups was not statistically significant (χ^2^ = 0.065, *P* = 0.798, Table 3). The age of the patients in the cVM group was 49.61 ± 9.03 years and the age of the patients in the pVM group was 44.52 ± 14.18 years. The age between the two groups showed a statistical difference (*t* = −2.578, *P* = 0.011, Table 3). There were 24 and 67 individuals with a history of motion sickness and 23 and 26 individuals without a history of motion sickness in the cVM and pVM groups, respectively. The results between the two groups were statistically different (χ^2^ = 6.04, *P* = 0.014, Table 3).

**TABLE 3:** Clinical data of the cVM and pVM groups.

	cVM group	pVM group	χ^2^/*T*-value	*P*-value
Gender (male/female)	11/36	20/73	0.065	0.798
Age (years)	49.61 ± 9.03	44.52 ± 14.18	2.578	0.011
Dizziness or vertigo (yes/no)	24/23	67/26	6.04	0.014

## Discussion

With the increasingly standardized diagnosis and treatment of vertigo and dizziness disorders, VM is receiving increasing attention from clinicians. Clinical work has identified many patients with VM who are undiagnosed during the first five episodes of dizziness and are eventually diagnosed with VM as the number of episodes increases over time. Therefore, the early diagnosis of patients with VM is a difficult problem that needs to be addressed in clinical work. Studies have shown that, there is auditory dysfunction at the lower frequencies in VM patients and the cochlear dysfunction of the peripheral auditory system (Xue et al., 2020). Besides, compared with migraine patients, the incidence of tinnitus was significantly increased in the VM group (Kirkim et al., 2017). Abnormal oculomotor functions are commonly observed in patients with VM (Fu et al., 2021). Previous studies have focused on the potential relationship and mechanism between migraine with vestibular symptoms and motion sickness. In our study, we have refined the classification, studies VM, and the cVM and pVM scales. Depicting their age distribution and differences in the incidence of motion sickness. Some studies have shown (Antal et al., 2005) that the visual cortex of migraine patients exhibits increased excitability, so the increase in motion perception may be caused by hyperexcitability of the visual cortex. Patients with VM have vestibular dysfunction with elevated perceptual thresholds, and their thresholds increase further after complex visual stimuli. Based on this finding, it has been hypothesized (Wurthmann et al., 2021) that the interaction between the visual and vestibular cortex may contribute to the occurrence of migraine and visual vertigo of VM, while during the interictal period, VM patients have reduced motor perception thresholds and show a marked susceptibility to motion sickness, which may indicate an increased level of vestibular processing. Previous studies (Winnick et al., 2018; Bednarczuk et al., 2019) have shown the presence of abnormal spatial location and sensory integration in patients with VM. Several other fMRI studies (Russo et al., 2014; Shin et al., 2014) have identified interactions between vestibular and visual cortical areas and activation of vestibular thalamocortical pathways during VM episodes. These findings could suggest that the occurrence of VM is related to vestibular, somatosensory, and visual information, and VM could be considered a complex disorder caused by multiple disorders of sensory integration. A small number of patients have reported visual aura, but more detailed data for analysis are difficult to obtain. Perhaps patients with aura have a higher prevalence of motion sickness, which deserves further investigation.

Various serum factors such as 5-hydroxytryptamine (5-HT), calcitonin gene-related peptide (CGRP), and Mg^2+^ have been found to be associated with VM (van Dongen et al., 2017; Wang et al., 2020; Zhang et al., 2020). Furthermore, some cytokines, such as interleukin-1β, CCL3, CCL22, and CXCL1, have also been found to be valuable in the diagnosis of VM (Flook et al., 2019). However, there is no single experimental index that can diagnose VM, serologically or with various cytokines, so it is not easy to find relatively specific indexes among many laboratory indexes. In some studies, subjective scales and motion sickness susceptibility questionnaires have reached similar conclusions, with more people susceptible to motion sickness in the migraine group than in the normal control group (Jeong et al., 2010). Similarly, we hope that in the future, motion sickness can be used as an accessible predictor to assist in the early diagnosis of VM.

Clinically relevant influences are more easily accessible than laboratory indicators. Some triggers such as sleep deprivation, stress, irregular diet, flickering light, odor stimuli, food, weather changes, and menstrual cycle have been suggested to be present in patients with VM (Neuhauser et al., 2001; Baier et al., 2009; Andress-Rothrock et al., 2010). Several studies have shown that about half of patients with VM have a family history of the disease (Oh et al., 2001; Lee et al., 2008; Teggi et al., 2018; Huang et al., 2020). The prediction of models using clinical factors and laboratory indicators in patients with VM to diagnose VM has also been reported in the literature (Zhou et al., 2020). However, fewer studies have been reported on the interrelationship between virtual reality and motion sickness. Previous studies have reported that compared with Meniere’s disease, benign paroxysmal positional vertigo or vestibular neuritis, migraine patients are more likely to have motion sickness. It is therefore hypothesized that the pathways involved in motion sickness and migraine are central rather than peripheral (Cuomo-Granston and Drummond, 2010). It has been suggested that although patients with VM have an increased susceptibility to motion sickness in general, this is not different from migraine (Murdin et al., 2015). However, other studies have further refined the symptoms of migraine patients, and migraine patients with dizziness/vertigo have more severe motion sickness than migraine patients without dizziness/vertigo and normal controls, which is similar to our results (Jeong et al., 2010). A study of motion sickness and VM showed that in a college student population, approximately half of those with motion sickness had VM in combination, while the prevalence of VM in those without motion sickness was only 30% (Abouzari et al., 2020), but the population involved in this study was in their 20s and was a motion sickness-centered study, making its clinical value relatively limited.

There are limited researches on the clinical differences between cVM and pVM, and some studies suggest that the patient groups’ characteristics were most pronounced for definite diagnoses (Oh et al., 2021), and there are other studies that support this conclusion, suggesting that utriculo-ocular pathway dysfunction is more frequent in VM than pVM (Fujimoto et al., 2020). In addition to these discussions between cVM and pVM, some studies suggest that the current criteria of VM is questionable and needs to be further refined and expanded (Dominguez-Duran et al., 2021; Chae et al., 2022). The results of this study showed that women with cVM were approximately 3–4 times more likely than male patients, and their age matched a normal distribution, with a high prevalence in middle-aged women around 50 years of age, which is consistent with previous studies (Dieterich and Brandt, 1999). However, the age distribution of pVM patients does not have an obvious age concentration trend and is distributed in all age stages, with a relatively higher prevalence in young and middle-aged women. The age distribution of patients with pVM, which is rarely reported in previous studies, may be related to the fact that the clinical diagnosis of patients with VM requires longer follow-up and the increased frequency of attacks near menopause and the presence of other conditions that are consistent with the diagnosis, so that the age would tend to be around 50 years. In contrast, pVM, because it requires relatively few conditions for diagnosis, requires a shorter follow-up period, and is diagnosed when patients are much younger. Previous statistical analysis shows a significant increase in the diagnosis of pVM with increasing age and a decrease in VM diagnosis. The type of vestibular symptoms varies according to the age of onset of VM. This conclusion may have been limited by the sample size and the investigators set a temporal cut-off of 60 years old, based on the evidence of the radical decrease in the onset of migraine attacks from the age of 50 (Ori et al., 2020). Furthermore, this study also found that patients with pVM had a higher rate of motion sickness, 72.04%, compared to 51.06% of patients in the cVM group and 23.00% in the control group. cVM patients with motion sickness were consistent with the study by Wurthmann et al. (2021). However, motion sickness in pVM patients as well as in the normal control population was also investigated in this study, and age and sex matched analysis revealed a statistically significant comparison between the proportion of the VM group and the physical examination group with a history of motion sickness.

Migraine is considered to be a neurological disorder. Due to the unpredictability of migraine and the lack of reliable and acceptable ways to trigger a typical migraine, it is difficult to examine patients during migraine attacks, especially to detect subtle neuronal activity or neurotransmitter levels (Marcus et al., 2005). However, motion sickness provides a reproducible phenomenon, allowing easy study in the research laboratory. Our research suggests a high correlation between motion sickness and VM, which can be used as one of the clinical predictors of early onset in patients with VM. Some studies believe that the diagnosis of motion sickness diagnosis is mainly clinical, based on the history of a triggering situation (imposed or perceived motion) and typical symptoms and signs of motion sickness such as nausea, headache, blurred vision, non-vertiginous dizziness, drowsiness, spatial disorientation, difficulty concentrating, and sometimes vomiting., etc. The diagnosis can be facilitated if there is a prior history of motion sickness, especially following the exposure to similar events, while laboratory testing is usually not necessary (Leung and Hon, 2019). It is worth noting that in studies of EDA (electrodermal activity), the participants were divided into groups based on whether they had Graybiel scores of 0 or ≥1 for all seven Graybiel diagnostic criteria (nausea, skin color, cold sweating, increased salivation, drowsiness, pain, and central nervous system). And the results showed that there was no difference in scores of 0 and ≥1 for autonomic symptoms except cold sweat and salivation, which suggested that self-reports may not be as accurate as we expect (Tamura et al., 2018). In 2021, signs and symptoms related to motion accounted for the majority of the diagnostic conditions in the Barany criteria. However, there have been studies evaluating bodily stability in different diseases using direct quantitative measures of movement (Cohen et al., 2014; Lubetzky et al., 2021). The quantitative kinematics of postural activity differ between persons who are susceptible to motion sickness, and those who are not. These differences exist during exposure to nauseogenic motion stimuli but, critically, also exist in the absence of such exposure. That is, measures of “ordinary body sway” can be used to predict the risk of motion sickness for individuals (Koslucher et al., 2016; Weech et al., 2018). The same effect has been observed in the case of mild traumatic brain injury (Chen et al., 2013). These indicators make the diagnosis of motion sickness seem more traceable than the signs and symptoms in the diagnostic criteria, and may further predict VM. Criteria was introduced in the manuscript to diagnose cVM and pVM, and according to the inclusion and exclusion criteria, the included patients with cVM and pVM were consistent with moderate or severe intensity, lasting 5 min to 72 h. Although the difference between cVM and pVM is the migration history or migration features during the episode in the criteria, it should be noted that the severity and duration of symptoms (dizziness and headache) may interfere with outcomes. In the future, we will also combine other influencing factors to conduct multifactorial impact analysis and model construction to guide VM for early clinical diagnosis. In addition, there are a series of new directions on the diagnosis and treatment of VM, including functional magnetic resonance imaging (fMRI), combined treatment with different antidepressants, which provide inspiration for the rehabilitation and prevention of vestibular function, and we will carry out a variety of new studies related to VM to further explore these contents. Most of episodic or progressive syndromes show familial clustering (Roman-Naranjo et al., 2018). To explore the familial genetics of VM, we established the Vertigo Diagnosis and Treatment Consortium. As a large public hospital, we work closely with the county hospitals and community hospitals to provide them with equipment and share technology. At present, these processes are still being improved, and we hope to collect more data and conduct multicenter studies on VM family history in the future.

## Data availability statement

The raw data supporting the conclusions of this article will be made available by the authors, without undue reservation.

## Ethics statement

The studies involving human participants were reviewed and approved by the Ethics Committee of the Affiliated Hospital of Jiaxing University. Written informed consent for participation was not required for this study in accordance with the national legislation and the institutional requirements.

## Author contributions

JH designed and developed this study and performed the analysis. DM and JH drafted and edited the manuscript. JZ and TH contributed to conception of the study. XZ, TJ, and HG collected the data. DM performed the interpretation. All authors contributed to the article and approved the submitted version.

## Footnotes

1
https://sample-size.net/
